# Discovery of Antibacterial Compounds with Potential Multi-Pharmacology against Staphylococcus *Mur ligase* Family Members by In Silico Structure-Based Drug Screening

**DOI:** 10.3390/molecules29163792

**Published:** 2024-08-10

**Authors:** Mio Teshima, Kohei Monobe, Saya Okubo, Shunsuke Aoki

**Affiliations:** Department of Bioscience and Bioinformatics, Graduate School of Computer Science and Systems Engineering, Kyushu Institute of Technology, Iizuka 820-8502, Japan

**Keywords:** MurE, *Staphylococcus aureus*, in silico structure-based drug screening (SBDS), docking simulation, molecular dynamics simulation

## Abstract

*Staphylococcus aureus* (*S. aureus*) is a major bacterial infection in humans, leading to severe disease and causing death. The stagnation of antibiotic development in recent decades has made it difficult to combat drug-resistant infections. In this study, we performed an in silico structure-based drug screening (SBDS) targeting the *S. aureus* MurE (saMurE) enzyme involved in cell wall synthesis of S. aureus. saMurE is an enzyme that is essential for the survival of *S. aureus* but not present in humans. SBDS identified nine saMurE inhibitor candidates, Compounds **1**–**9**, from a structural library of 154,118 compounds. Among them, Compound **2** showed strong antibacterial activity against *Staphylococcus epidermidis* (*S. epidermidis*) used as a model bacterium. Amino acid sequence homology between saMurE and *S. epidermidis* MurE is 87.4%, suggesting that Compound **2** has a similar inhibitory effect on *S. aureus*. Compound **2** showed an IC_50_ value of 301 nM for *S. epidermidis* in the dose-dependent growth inhibition assay. Molecular dynamics simulation showed that Compound **2** binds stably to both *S. aureus* MurD and *S. aureus* MurF, suggesting that it is a potential multi-pharmacological pharmacological inhibitor. The structural and bioactivity information of Compound **2**, as well as its potential multiple-target activity, could contribute to developing new antimicrobial agents based on MurE inhibition.

## 1. Introduction

*Staphylococcus aureus* (*S. aureus*) is one of the most common pathogens of bacterial infections in humans, resulting in various serious symptoms, including bacteremia, infective endocarditis, and skin and soft tissue diseases [1,2]. Vancomycin-resistant *S. aureus* (VRSA) and methicillin-resistant *S. aureus* (MRSA) have emerged and spread globally. MRSA has acquired resistance to many antibiotics used in clinical practice, making it extremely difficult to treat infections caused by MRSA [3]. In the past, infections caused by MRSA were prevalent only in medical facilities; however, community-acquired MRSA, which can be transmitted to healthy people, has emerged in recent years and has become a severe problem. The development of new antibiotics needed to combat antibiotic-resistant infections is inadequate, and new antimicrobial agents need to be developed and delivered to clinical practice [4].

Peptidoglycans, which account for about 95.0% of the cell wall in Gram-positive bacteria, are mainly composed of cross-linked structures of N-acetylglucosamine and N-acetylmuraminic acid [5,6]. Members of the Mur ligase family are specifically involved in the process of peptidoglycan biosynthesis in the cell walls of various bacteria. UDP-N-acetylmuramoyl-L-alanyl-D-glutamate-2,6-diaminopimelate ligase (MurE) catalyzes the reaction linking meso-diaminopimelic acid (m-A2pm) to UDP-N-acetylmuramoyl-L-alanyl-D-glutamate acid (UDP-MurNAc -L-alanyl-D-glu). On the other hand, MurE catalyzes the reaction linking L-Lys instead of m-A2pm to the substrate in Gram-positive bacteria [7,8]. This reaction catalyzed by MurE is essential for cell wall synthesis in *S. aureus*, and inhibition of MurE function leads to inhibition of bacterial growth and lysis due to loss of osmotic resistance. In a previous study, a small-molecule *S. aureus* MurE (saMurE) inhibitor with carboxyl groups was found, and the small-molecule compound inhibited saMurE and showed antibacterial activity [9,10]. However, no small-molecule inhibitory compounds targeting saMurE exist as medical drugs, and none are currently in clinical trials. saMurE has been crystallized, and its three-dimensional structure has been elucidated by X-ray structural analysis.

In silico structure-based drug screening (SBDS) is a computational approach to drug discovery that allows for fast and low-cost discovery of useful compounds in a vast chemical space. In recent years, research on new antimicrobial compounds against infectious diseases has seen a rapid increase in the use of structure-based approaches, leading to the identification of many beneficial compounds [11,12]. Hierarchical in silico SBDS combining docking simulations with molecular dynamics simulations has successfully identified useful antimicrobial compounds against *Staphylococcus* bacteria [11]. Molecular dynamics simulation (MDS) is a method to predict atomic interactions from the motion of all atoms in a system composed of proteins, compounds, etc. Docking simulation (DS) is computationally inexpensive and enables high-speed screening of many compounds. On the other hand, MDS can capture a wide variety of important biomolecular processes, such as changes in conformational, protein folding, and ligand binding, and reveal their behavior with the time axis. Furthermore, the binding free energy can be evaluated using enthalpy and entropy, resulting in high binding prediction accuracy. MDS has the problem of extremely high computational cost compared to DS. Constructing hierarchical screening systems that balance these two factors in terms of computational cost and accuracy improved drug screening performance [12].

In this study, a hierarchical in silico SBDS with a balanced combination of DS and MDS was performed to identify antimicrobial compounds with a novel scaffold targeting saMurE. As a result, an anti-Staphylococcus compound with a characteristic skeleton with an IC_50_ value of 301 nM against saMurE was identified. Furthermore, MDS analyses revealed the potential multi-pharmacological activity against MurD, MurE, and MurF. Our compounds are important candidates with a promising future in the development of antimicrobial agents for MRSA infections.

## 2. Results

### 2.1. Hierarchical In Silico SBDS Targeting saMurE

**Figure 1:**
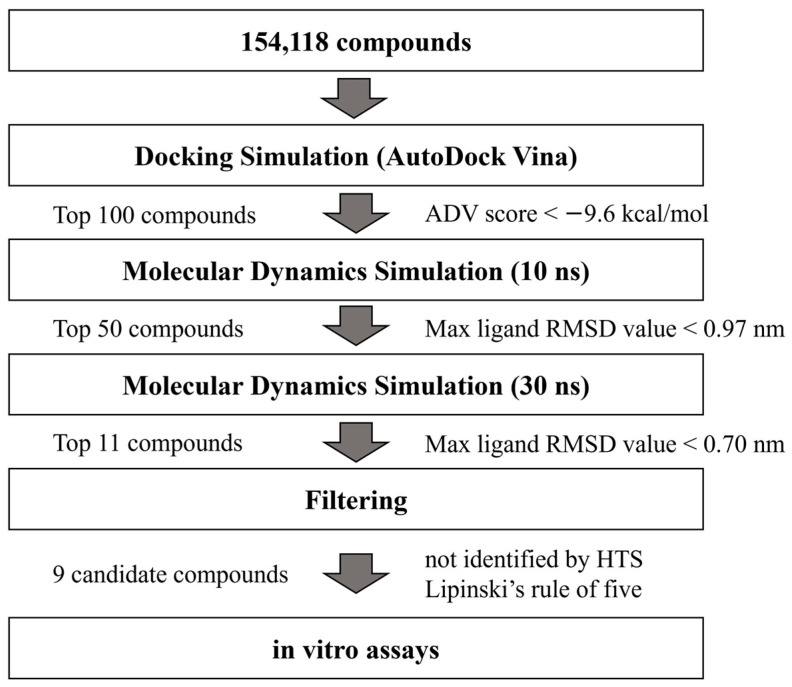
saMurE inhibitor screening strategy.

Hierarchical in silico SBDS targeting saMurE was performed using the ChemBrige 3D structure library of 154,118 compounds (Figure 1). Flexible docking simulations (ligand only) with AutoDock Vina (ADV) [13] selected 100 compounds with ADV scores less than −9.6 kcal/mol. The docking poses of 100 compounds predicted by ADV were used as the initial structures, and MDSs of 10 ns time frame were performed after minimization and equilibration processes. Fifty compounds with a maximum ligand root mean square deviation (RMSD) value of less than 0.97 nm were selected for MDSs of 30 ns time frame. Compounds that did not conform to Lipinski’s rule of five and compounds that have been bioassayed against *S. aureus* were eliminated. Nine compounds with a maximum ligand RMSD value of less than 0.70 nm were selected as final candidates. ChemBridge ID, IUPAC name, and maximum ligand RMSD values of 30 ns MDSs are shown in Table S1.

### 2.2. Growth Inhibition Assay against Staphylococcus epidermidis (S. epidermidis)

**Figure 2:**
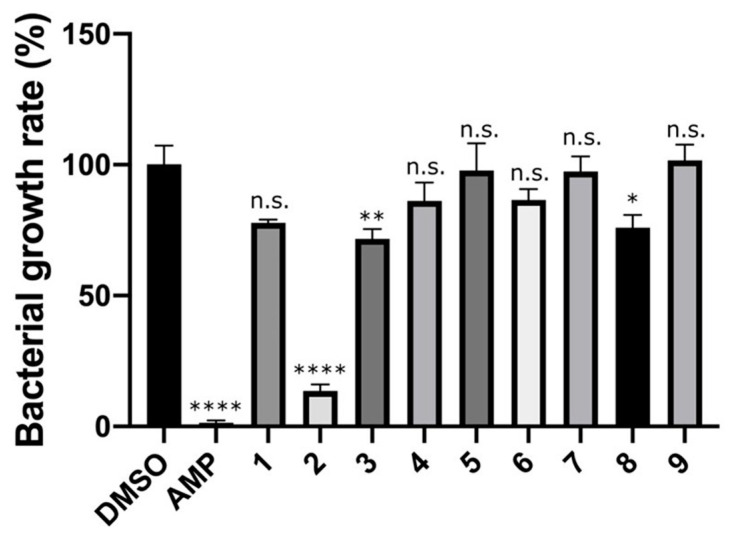
Growth inhibitory effect of Compounds **1**–**9** on bacteria (*S. epidermidis*). A total of 0.3% DMSO and 100 μM ampicillin (AMP) were used as samples for comparison. Compounds **1**–**9** (100 μM). The vertical axis is the mean +/− SEM of the results of four independent experiments. Dunnett’s test: ****; *p* < 0.0001; **; *p* < 0.0021; *; *p* < 0.0332; n.s. = not significant.

**Figure 3:**
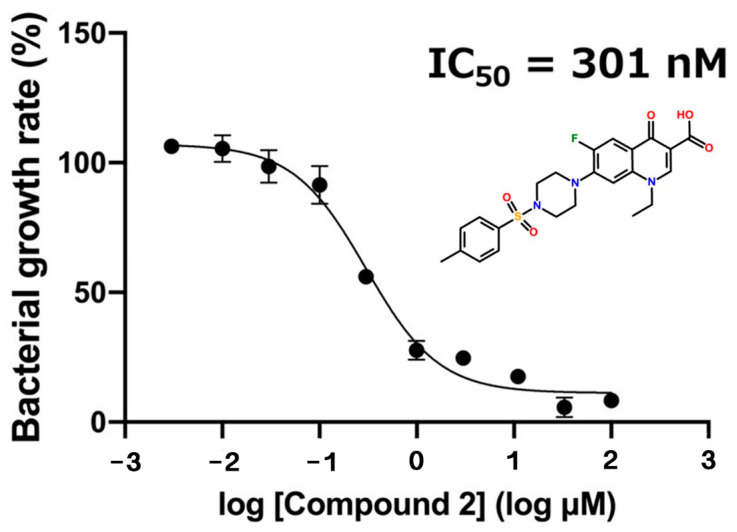
Determination of 50% growth inhibition concentration against bacteria (*S. epidermidis*). The vertical axis shows the relative bacterial growth rate. The horizontal axis shows the molar concentration of Compound **2**.

Nine candidate compounds (Compounds **1**–**9**) selected by in silico SBDS were subjected to growth inhibition assays against *S. epidermidis*. *S. epidermidis* was used because it is not permitted to conduct experiments using *S. aureus*, which is a biosafety level 2 organism. The BLAST server analyzed the amino acid sequence between saMurE and *S. epidamidis* MurE (seMurE). The amino acid sequence homology between saMurE and seMurE is 87.4%. The results of the growth inhibition assay showed that among the nine candidate compounds, Compound **2**, Compound **3,** and Compound **8** inhibited the growth of *S. epidermidis* (Figure 2). The growth inhibition rates against *S. epidamidis* of Compound **2**, **3,** and **8**, for which significant differences were identified, were 86.4%, 28.3%, and 24.1%, respectively. The dose-dependent effect of Compound **2** on the growth of *S. epidermidis* was examined, and the 50% growth inhibition concentration (IC_50_ value) was calculated, resulting in an IC_50_ value of 301 nM for Compound **2** (Figure 3).

### 2.3. MDS Trajectory Data Analysis of the saMurE–Compound ***2*** Complex

**Figure 4:**
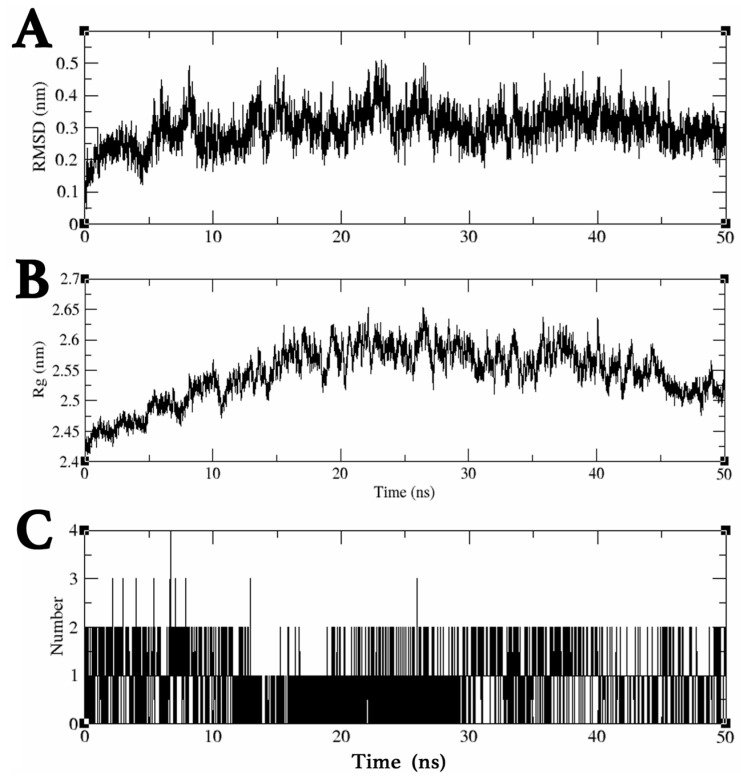
MDS results for the saMurE–Compound **2** complex: (**A**) Transition of ligand RMSD value (nm). Ligand RMSD values were calculated by comparison with the post-equilibration pose. (**B**) Radius (nm) of gyration during MDS. (**C**) Number of intermolecular hydrogen bonds.

The dynamic behavior of the Compound **2** and saMurE complex was analyzed by MDS in a 50 ns time frame to assess ligand binding stability. Trajectory data obtained by MDS were analyzed using ligand RMSD, gyrate, and hbond modules. The mean and maximum ligand RMSD values for Compound **2** were 0.30 nm and 0.51 nm, respectively, confirming the stable binding of Compound **2** to the active site of saMurE (Figure 4A). The complex structure around 25 ns, when the ligand RMSD value reached its maximum level, was confirmed by the molecular visualization software Visual Molecular Dynamics (VMD) [14], which confirmed that Compound **2** was bound to the active site of saMurE. The average and maximum radii of rotation of the Compound **2**–saMurE complex were 2.54 nm and 2.65 nm, respectively, confirming that the folding structure of saMurE was preserved (Figure 4B). The number of hydrogen bonds between saMurE and Compound **2** was one to two during MDS (Figure 4C).

### 2.4. Analysis of Binding Modes and Binding Free Energies of saMurE and Compound ***2***

**Figure 5:**
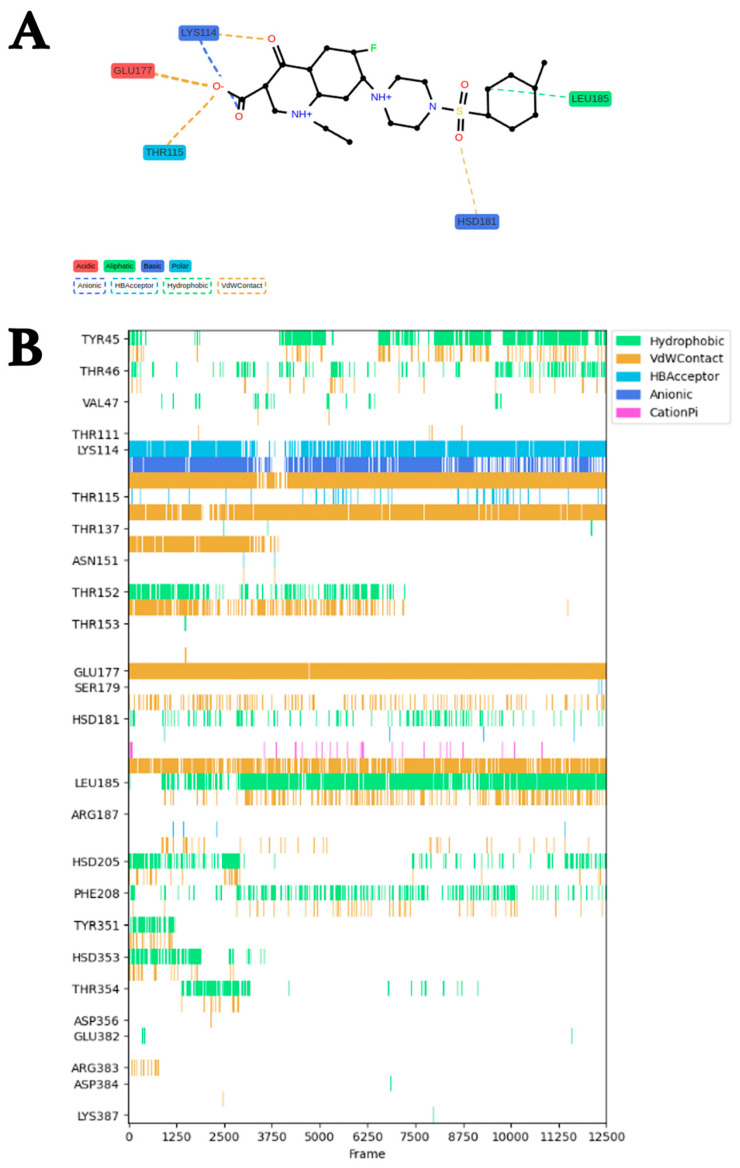
ProLIF analysis for the saMurE–Compound **2** interaction: (**A**) Interacting residues throughout the MDS timeframe. In the screening process, the piperazine group of Compound **2** is protonated. (**B**) A major (≥60% probability of presence) interaction residue group was observed throughout the entire period.

ProLIF [15] was used to analyze the molecular interactions between saMurE and Compound **2**. Fingerprint analysis of trajectories obtained by a 50 ns MDS of saMurE and Compound **2** predicted the interacting amino acid residues, the functional groups of the compounds, and the molecular interaction types. Various molecular interactions were observed between 25 amino acids of saMurE and Compound **2** from 0 ns to 50 ns (Figure 5A). Five amino acid residues, Lys114, Thr115, Leu185, Hsd181, and Glu177, formed interactions with Compound **2** in 60% of the entire MDS timescale. Of those five amino acids, Leu185 was involved in hydrophobic interactions, Lys114 in electrostatic interactions, and the others in van der Waals interactions (Figure 5B). Lys114 was frequently engaged in hydrogen bonding and van der Waals interactions as well as electrostatic interactions. The binding free energy analysis was performed using ab initio fragment molecular orbital (FMO) [16] calculations, and the binding free energy between Compound **2** and saMurE was −61.1 kcal/mol.

### 2.5. Growth Inhibition Assay for Gram-Negative Bacterium

The antibacterial effect of Compound **2** on *Escherichia coli* (*E. coli*) (BL21 strain), a Gram-negative bacterium, was verified. Although a homology search by BLAST showed that the amino acid sequence homology between saMurE and *E. coli* MurE was 31.0%, Compound **2** (100 μM) showed moderate antibacterial activity against *E. coli* with a growth inhibition rate of 73.9% (Figure S1).

### 2.6. Prediction of Pharmacological Properties and Toxicity of Compound ***2***

The SwissADME web server [17] was used to predict drug similarity and pharmacological affinity of Compound **2**. In all six indicators of SwissADME (lipophilicity, molecular weight, polarity, solubility, saturation, and flexibility), the molecular descriptor parameters of Compound **2** remained within the appropriate range, confirming its suitability as a drug (Figure S2). The results of the prediction of toxicity of Compound **2** using the ProTox-II web server [18,19] showed that 16 of the 17 predicted toxicity items were non-toxic, while hepatotoxicity was alerted (Figure S3).

### 2.7. Toxicity Assays for Mammalian-Derived Cells

**Figure 6:**
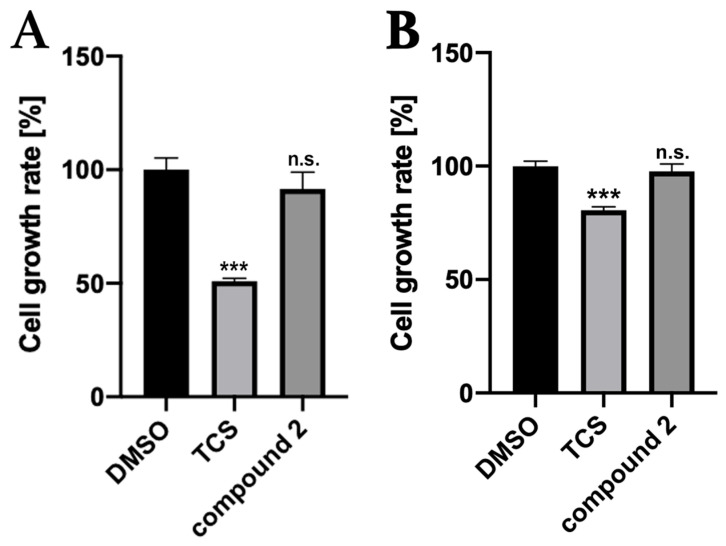
Toxicity verification of Compound **2** on mammalian-derived cells: (**A**) COS-7 cells; (**B**) HepG2 cells. Negative control was 0.3% DMSO and positive control was 50 μM triclosan (TCS). Concentration of Compound **2** was 100 μM. Dunnett’s test: ***; *p* < 0.0002; n.s. = not significant.

Toxicity assays for Compound **2** were performed in two types of cells derived from mammals, including humans. Compound **2** was not toxic to COS-7 cells derived from African green monkey kidneys at a concentration of 50 μM. On the other hand, it showed no toxicity to human liver-derived HepG2 cells. Triclosan, commonly known as an antimicrobial agent, was toxic to both cells (Figure 6).

### 2.8. Membrane Permeation Simulation of Compound ***2*** against Gram-Positive Bacteria

Umbrella sampling using lipid bilayers composed of the monomer 6-O-methacryloyl-1,2;3,4-di-O-isopropylidene-d-galactopyranose (MAIPG) [20], one of the plasma membrane constituent lipids of Gram-positive bacteria [21], was performed to investigate in silico the energy gradient generated during cell membrane permeabilization of Compound **2** (Figure S4). The relative energy to the free energy of Compound **2** when its surroundings are filled with water molecules is found to increase as it moves perpendicular to the lipid bilayer. Compounds that exhibit such energy profiles are likely to be membrane-permeable, since the compounds whose free energy in the lipid bilayer is lower than the free energy in water molecules are expected to remain in the lipid bilayer and have lower membrane permeability.

### 2.9. Interaction Analysis of Compound ***2*** and Other Members of the Mur ligase Family by MDSs

MurC, MurD, MurE, and MurF have protein structures similar to each other and are enzymes that make sequential additions of amino acids and dipeptides to UDP-MurNAc, which is essential for peptidoglycan synthesis. The basic structure of the substrates of these enzymes is based on UDP-MurNAc, and similarities of MurC, MurD, and MurF substrates to UDP-MurNAc were compared using the Tanimoto coefficient, which was 0.936, 0.928, and 0.926, respectively. Thus, Compound **2** possibly binds to other *S. aureus* Mur (saMur) ligases. DSs of Compound **2** were performed for each *S. aureus* MurC (saMurC), *S. aureus* MurD (saMurD), and *S. aureus* MurF (saMurF), and a 50 ns MDS was performed with the resulting complex as the initial structure. The three-dimensional structure of saMur family members was obtained from AlphaFold2 [22].

### 2.10. MDS Trajectory Analysis of Compound ***2*** and MurC, MurD, and MurF Complexes

**Figure 7:**
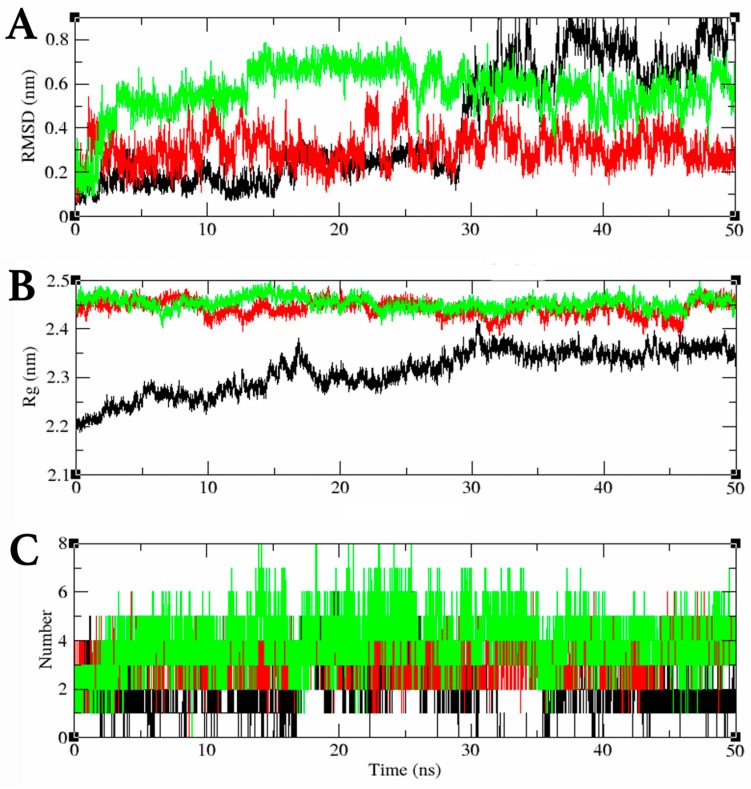
MDS results for saMur ligase–Compound **2** complexes: (**A**) ligand RMSD values (nm); (**B**) radius (nm) of gyration during MDS; (**C**) number of intermolecular hydrogen bonds. saMurC (black), saMurD (red), saMurF (green).

In the MDS of the saMurC–Compound **2** complex, the ligand RMSD value of Compound **2** remained close to 0.2 nm from 0 ns to around 30 ns, and the ligand RMSD value rapidly increased after 30 ns, exceeding 1.2 nm, and Compound **2** was dissociated from the active site of saMurC. In the MDS of the saMurD–Compound **2** complex, the ligand RMSD value of Compound **2** ranged between 0.2 nm and 0.4 nm, indicating binding at the active site of MurD throughout the 50 ns MDS. In the MDS of the saMurF–Compound **2** complex, the ligand RMSD value of Compound **2** ranged between 0.1 nm and 0.6 nm, indicating binding at the active site of MurD throughout the 50 ns MDS (Figure 7A). Both saMurD and saMurF turnover radii remained between 2.4 nm and 2.5 nm throughout the MDSs, confirming stable folding of the proteins over 50 ns of MDSs (Figure 7B). The saMurD–Compound **2** complex formed a maximum of 6 and an average of 3.4 hydrogen bonds during the MDS, and the saMurF–Compound **2** complex formed a maximum of 8 and an average of 4 hydrogen bonds during the MDS (Figure 7C).

## 3. Discussion

### 3.1. In Silico SBDS Constructed by Hierarchical Combination of DS and MS

In this study, we succeeded in highly efficient drug identification by performing in silico SBDS, which was constructed by hierarchically combining DS and MDS, and this method will be important as a new method for drug discovery in the future. The area under the curve (AUC) value calculated from receiver operator characteristic curve analysis by evaluating the binding stability from the ligand RMSD value of the compound calculated using trajectories obtained from the MDS is significantly increased [23]. In this method, 9 compounds were selected from a library of approximately 150,000 compounds using the ligand RMSD value as an indicator, and the hit rate was 33% (3 hits/9 compounds), successfully identifying compounds that showed antibacterial effects at 301 nM. The combination of DS followed by MDS and evaluation by ligand RMSD was a very effective method. In previous studies, new compounds with high antimicrobial activity were also identified in SBDS using ligand RMSD values targeting *Staphylococcus aureus* methionyl-tRNA synthetase [11].

### 3.2. Antimicrobial Spectrum of Compound ***2***

Compound **2** inhibited *S. epidermidis* growth by 86.4% at 100 μM, with an IC_50_ value in the nanomolar range of 301 nM. Compound **2** inhibited the growth of Gram-positive *Mycobacterium* sp. and Gram-negative *E. coli* by 82.0% and 73.9%, respectively, indicating that it has a relatively broad antimicrobial spectrum (Figure S5). The antibacterial activity of Compound **2** against bacteria other than *Staphylococcus* was relatively weak. Since MurE is not present in humans but in a wide variety of bacteria, the antimicrobial spectrum could be expanded by searching for structures similar to Compound **2**. Although several inhibitor development studies targeting saMurE have been reported in the past [9,10], this is the first discovery of a compound that shows antibacterial efficacy against saMurE at the nanomolar range by in silico SBDS, a hierarchical combination of DS and MDS. Furthermore, the Compound **2** skeleton discovered in this study has great promise as a platform for new drugs to combat MRSA and VRSA.

### 3.3. Potential Multiple Pharmacological Properties of Compound ***2***

In the MDS, the mean and maximum ligand RMSD values of Compound **2** for binding to saMurD were 0.31 nm and 0.61 nm, respectively. Furthermore, the mean and maximum ligand RMSD values for saMurF were 0.57 nm and 0.81 nm, respectively, suggesting that Compound **2** has potential binding properties not only for saMurE but also for saMurD and saMurF. These data support the possibility that Compound **2** has potential multi-pharmacological pharmacological properties. Compound **2** is anticipated to have high resistance to the emergence of resistant strains due to its potential multi-pharmacological pharmacological activity.

### 3.4. In Silico Toxicity Prediction and In Vitro Cytotoxicity Assay Using Mammalian-Derived Cells

Although the machine learning toxicity prediction tool Protox-II predicted non-toxicity for 16 classifications of human toxicities, Protox-II warned of weak hepatotoxicity with a safety probability of less than 0.6. In vitro, cytotoxicity experiments on kidney-derived COS-7 cells and liver-derived HepG2 cells showed no toxicity. These combined in silico and in vitro data suggest that Compound **2** has specific antimicrobial activity against *S. aureus* but is not toxic to mammals. However, the hepatotoxicity predicted by machine learning tools in silico should be carefully validated in the future.

### 3.5. Major Group of Amino Acid Residues That Support Stable Binding of Compound ***2***

Interaction residue analysis by ProLIF indicated that Lys114, Thr115, Leu185, Hsd18, and Glu177 are crucial residues for binding between saMurE and Compound **2**. During MDS, Lys114 was frequently involved in hydrogen bonding and van der Waals interactions as well as electrostatic interactions, indicating that it is a key anchor amino acid residue in establishing stable binding with Compound **2**. These amino acid residues were found to interact intensively with the oxoquinoline carboxylic acid portion of Compound **2**. In particular, the electrostatic interaction formed by the negative charge by deprotonation of the oxoquinoline carboxylic acid moiety and the positive charge of the amino moiety of Lys114 would promote the binding stability of Compound **2**. Information on these molecular interactions is expected to support future in silico design and discovery for more pharmacologically active analogues.

### 3.6. Diversity of Compound ***2*** Targets

We have conducted in silico drug discovery studies using Mur ligases as target molecules, and Compound **2**, a ciprofloxacin analog, was identified. Many ciprofloxacin derivatives are generally known as antimicrobial agents targeting bacterial DNA gyrases. On the other hand, a group of compounds almost identical to Compound **2** was reported to have antimicrobial effects by Nieto et al. in 1999 [23]. However, the target molecules of those antimicrobial compounds have long been unknown. We have shown for the first time that this compound is a potential inhibitor targeting multiple Mur ligases (MurE, MurD, and MurF). Future in vitro biochemical experiments should confirm whether Compound **2** has inhibitory activity against DNA gyrases and Mur ligases.

## 4. Materials and Methods

### 4.1. Compound Structure Data Library

The compound structure data library used in this study is the ChemBridge 3D structure library of 154,118 compounds obtained from the RPBS (Ressource Parisienne en Bioinformatioque Structurale) web-based database. This compound structure data library is filtered by the ADME/Tox (absorption, distribution, metabolism, excretion, toxicity) filter to remove compounds unsuitable for pharmaceutical use. The compounds are ionized under pH7 conditions.

### 4.2. Pretreatment of Target Proteins

The steric structure of saMurE was obtained from the Protein Data Bank (PDB ID: 4C13) [24]. Water molecules, ions, and substrates were removed from the crystal structure using Molecular Operating Environment (MOE) 2019.0102. Hydrogen atoms were added to the three-dimensional structure of saMurE with the protonate 3D module of the MOE. The partial charge module was used to add charge, and the energy minimize module was used to minimize the energy. Molecular surface extraction and pocket search were performed using the DMS and sphgen programs of UCSF Chimera, respectively [25]. In silico SBDS was performed with the substrate binding sites of saMurE. Structural data of saMurC, saMurD, and saMurF were obtained from the AlphaFold2 database (A8Z4G7, Q2FZ92, and A0A0H2WWP1). These were processed and simulated in the same process as saMurE. The radius of gyration of the protein was calculated using the gyrate module to examine the conformational changes in the protein.

### 4.3. Docking Simulation

Docking simulations were performed using Auto Dock Vina 1.1.2 (ADV) [13] for the substrate binding sites of MurE with the compound 3D structure library (154,118 compounds) from ChemBridge. The scoring function for ADV consists of two Gaussian functions, a repulsive term, a hydrogen bonding term, a hydrophobic term, and a rotatable bond number. Flexible docking simulations for ADV were based on genetic algorithms.

### 4.4. Molecular Dynamics Simulation

MDS was performed on the protein–compound complex predicted by docking simulations to assess the stability of compound binding to the active site of MurE. MDS was performed using GROMACS 2023.2 [26]. Systems consisting of protein–compound complexes, water molecules, and ions were constructed with the Solution Builder on the CHARMM-GUI web server [27,28]. The simulation system used the CHARMM36m [29] force field. Cubic boxes were constructed, solvated with TIP3P water molecules, and neutralized with sodium and chloride ions at a salt concentration of 0.15 M. The energy minimization of the system was performed in the cubic box. The energy minimization was performed in up to 5000 steps using the steepest descent method. Equilibration was performed in two steps under NVT and NPT conditions, and the simulation environment was adjusted to a temperature of 310 K and a pressure of 1 bar. The LINCS algorithm was used to constrain the covalent bonding of hydrogen atoms [30]. The cutoff values for electrostatic and van der Waals interactions were determined to be 1.2 nm. Long-range electrostatic interactions were calculated using the particle mesh Ewald method [31,32]. Production MD was performed in 2 fs time steps.

### 4.5. MDS Trajectory Data Analysis

The ligand RMSD values of the compounds were calculated using the rms module of GROMACS to elucidate the binding stability of the compounds to the target proteins. The deviation from its binding position was plotted on the graph as ligand RMSD values using Grace. The number of hydrogen bonds between proteins and compounds was calculated using the hbond module to determine the contribution of hydrogen bonds to binding stability. Guterres et al. report that ligand RMSD values derived from MD trajectory data can be used to assess target binding capacity by small-molecule compounds (AUC value = 0.832) [33].

### 4.6. Umbrella Sampling

The simulation system, consisting of water, lipid bilayers, and compounds, was built with Membrane Builder of a CHARMM-GUI web server [32,34]. The free energy of Compound 2 across the MAIPG lipid bilayer [20], a membrane model of Gram-positive bacteria, was calculated from the potential of mean force (PMF) [25] using the umbrella sampling method [21]. MD simulation conditions were set up to draw molecules from the aqueous phase into the opposite membrane [35]. Seven hundred configurations were generated along the *Z*-axis direction (reaction coordinates). Each window was adjusted within 0.2 nm of the next window. Equilibration was performed at 100 ps in NPT conditions, followed by a 10 ns simulation. PMF profiles were obtained by the weighted histogram analysis method (WHAM) [36] using the wham module of GROMACS.

### 4.7. Preparation of Compounds

Compounds **1**–**9** were purchased from ChemBridge and dissolved in dimethyl sulfoxide (DMSO, Sigma) to 100 µM.

### 4.8. Bacterial Growth Inhibition Assay

*S. epidermidis* was obtained from the Microbial Materials Development Office, RIKEN BioResource Center (Saitama, Japan). *S. epidermidis* stock solution was added to 3 mL of culture medium [1% peptone (BD), 1% beef extract (BD), 0.5% NaCl (Wako, Japan), adjusted to pH 7.0] and incubated at 37 °C, 240 rpm for 2 h. The bacterial culture medium was diluted 20-fold and 0.3% DMSO (negative control), ampicillin (positive control), and candidate compounds were added to each well of a 96-well plate. The bacterial cells were incubated at 37 °C, 240 rpm for 6 h, and turbidity (OD595) was measured using a microplate reader (Bio-Rad Laboratories, Inc., Hercules, CA, USA).

Stock solution of *E. coli* (strain BL21) was added to 3 mL of culture medium [0.5% yeast extract (BD), 0.5% NaCl (Wako), 1% tryptone (BD), pH 7.0] and incubated at 37 °C 240 rpm for 24 h. The culture medium was diluted 20-fold and 0.3% DMSO (negative control), ampicillin (positive control), and Compound **2** were added to each well of a 96-well plate, incubated at 37 °C, 240 rpm for 8 h, and turbidity (OD595) was measured every 0 h, 4 h, and 8 h.

### 4.9. Toxicity Assays for Mammalian-Derived Cells

Mammalian-derived cells were cultured in 96-well plates. Cells were cultured in RPMI 1640 medium containing 5% fetal bovine serum, 100 U/mL penicillin, and 100 µg/mL streptomycin. Cells were cultured at 37 °C in 5% CO_2_. The medium was replaced with starvation medium, and the test compounds were added 24 h later. Triclosan and 0.3% DMSO were positive and negative controls, respectively. The cells were incubated for another 24 h, and cytotoxicity was tested using Cell Counting Kit-8 (Dojin, Kumamoto, Japan).

### 4.10. Ab Initio Fragment Molecular Orbital (FMO) Calculation

Ab initio FMO calculations were performed using ABINIT-MP6.0. The protein structure of saMurE was processed using the structure preparation module of MOE. For the binding free energy analysis of the interaction between saMurE and Compound **2**, fragmentation was performed using the abinitmp program. The theoretical level in the FMO calculation was set to MP2/6–31G. Biostation viewer 15.0 was used to calculate the IFIE of saMurE, and IFIEs for the compounds were calculated using Biostation viewer 15.0.

### 4.11. Statistical Analysis

All statistical analyses were performed using GraphPad Prism version 4 (GraphPad Prism software, Inc, San Diego, CA, USA).

## 5. Conclusions

In this study, in silico SBDS targeting saMurE was performed to identify compounds with antimicrobial activity against *S. epidermidis*. The IC_50_ value of the antibacterial activity of Compound **2** was 301 nM, and we succeeded in discovering a skeleton with extremely high activity by combining in silico and in vitro methods. The MDS results suggested that Lys114 of saMurE is a key anchor amino acid residue in the stable binding of Compound **2**. Furthermore, MDS was performed on other Mur ligase families, suggesting that Compound **2** potentially binds stably not only to saMurE but also to saMurD and saMurF. The major interacting amino acid residues of Compound **2** are intensively bound to the oxoquinoline carboxylate group of Compound **2**, suggesting that it is an important scaffold. Compound **2** is anticipated to have high resistance to the emergence of resistant strains due to its potential multi-pharmacological pharmacological activity and is expected to be a potent antimicrobial agent against MRSA and VRSA.

## Data Availability

The data presented in this study are available upon request from the corresponding author.

## Supplementary Materials

The following supporting information can be downloaded at https://www.mdpi.com/article/10.3390/molecules29163792/s1: Table S1: Characterization of candidate compounds and maximum ligand RMSD values; Table S2: Chemical structure of candidate compounds; Figure S1: Pharmacological effects of Compound **2** on E. coli; Figure S2: Chemical and pharmacological properties of Compound **2** predicted by SwissADME; Figure S3: Toxicity prediction of Compound **2** by ProTox-II; Figure S4: Free energy profile of Compound **2** for umbrella sampling; Figure S5: Growth inhibition rate against *Mycobacterium*.
