# Supplementary material for: Discovery of Antibacterial Compounds with Potential Multi-Pharmacology against Staphylococcus Mur ligase Family Members by In Silico Structure-Based Drug Screening
Source: Molecules. 2024 Aug 10;29(16):3792. doi: 10.3390/molecules29163792 (PMC11356833; doi:10.3390/molecules29163792)
Supplement: Supplementary file 1 [file molecules-29-03792-s001.zip › molecules-3104188-supplementary.pdf]

Table S1. Characterization of candidate compounds and maximum ligand RMSD values.

Name	ID	IUPAC	maximum ligand RMSD [nm]
Compound 1	7748081	2-[4-chloro-2-(1,8-dioxo-1,2,3,4,5,6,7,8,9,10-decahydro-9-acridinyl)phenoxy]-N-(4-chlorophenyl)acetamide	0.418
Compound 2	7036848	1-ethyl-6-fluoro-7-{4-[(4-methylphenyl)sulfonyl]-1-piperazinyl}-4-oxo-1,4-dihydro-3-quinolinecarboxylic acid	0.509
Compound 3	5353462	2-{[4-hydroxy-1-(2-methoxyphenyl)-6-oxo-1,6-dihydro-2-pyrimidinyl]thio}-N-1-naphthylacetamide	0.602
Compound 4	5766526	2-(1,3-benzothiazol-2-yl)-5-(4-oxo-4H-3,1-benzoxazin-2-yl)-1H-isoindole-1,3(2H)-dione	0.609
Compound 5	5224142	4-{[2-(3-ethynylphenyl)-1,3-dioxo-2,3-dihydro-1H-isoindol-5-yl]sulfonyl}benzoic acid	0.643
Compound 6	7159467	5-(1,3-dioxo-1,3,3a,4,7,7a-hexahydro-2H-isoindol-2-yl)-N-(4-methylphenyl)-2-(4-morpholinyl)benzamide	0.657
Compound 7	5186895	1,1',3,3'-tetraoxo-2'-phenyl-1,2',3,3'-tetrahydro-1'H-2,5'-biisoindole-5-carboxylic acid	0.671
Compound 8	7732114	3-[4-(1H-1,2,3-benzotriazol-1-ylcarbonyl)-1-phenyl-1H-pyrazol-3-yl]-2H-chromen-2-one	0.684
Compound 9	7109157	3-{[3-(3,4-dihydro-2(1H)-isoquinolinylsulfonyl)benzoyl]amino}benzoic acid	0.694

Table S2. Chemical structure of candidate compounds.

Name	ID	Chemical structure
Compound **1**	7748081	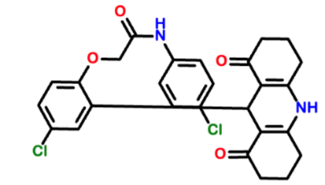
Compound **2**	7036848	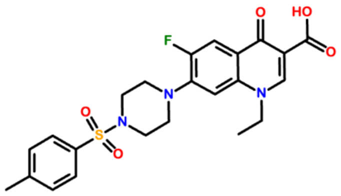
Compound **3**	5353462	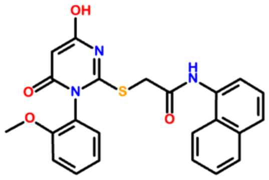
Compound **4**	5766526	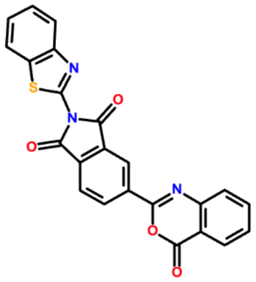
Compound **5**	5224142	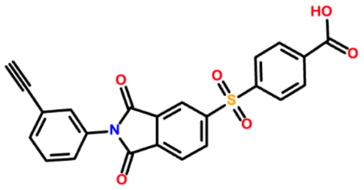
Compound **6**	7159467	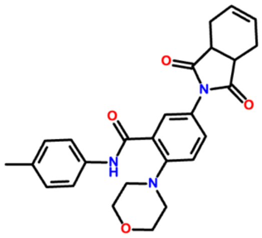

Compound **7**	5186895	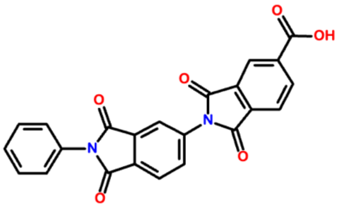
Compound **8**	7732114	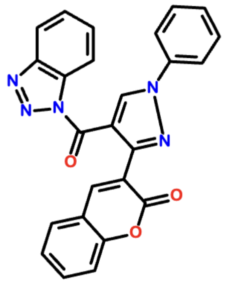
Compound **9**	7109157	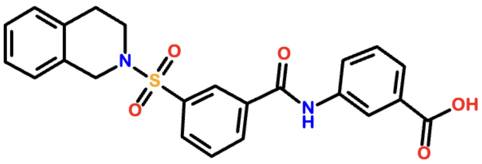

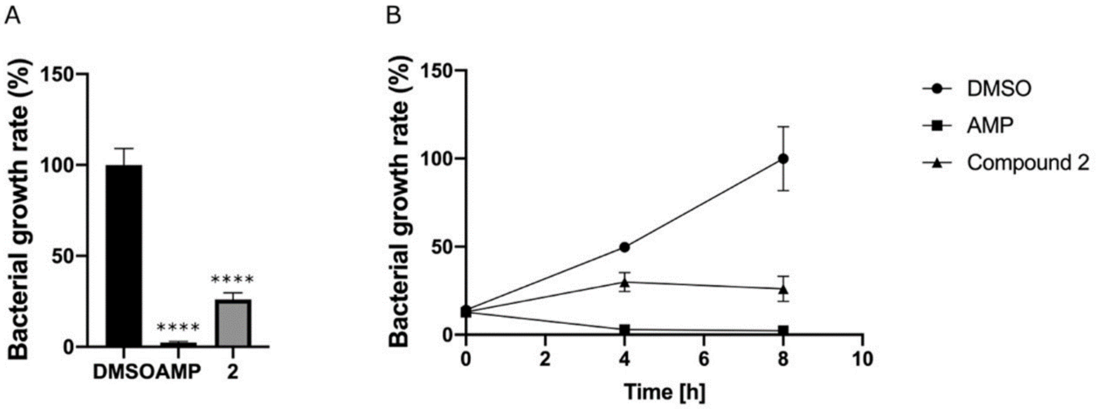

Figure S1. Pharmacological effects of Compound **2** on *E. coli*

(A) Growth inhibition rate against E. coli. (B) Change in bacterial growth over time. Negative control was 0.3% DMSO and positive control was 100 μM ampicillin (AMP). Concentration of Compound **2** is 100 μM. Dunnett's test: ****$p < 0.0001$.

Molecule 1

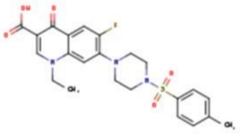

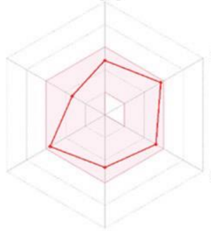

SMILES CCn1cc(C(=O)O)c(=O)c2c1cc(N1CCN(CC1)S(=O)(=O)c1ccc(cc1)C)c(c2)F

Physicochemical Properties	
Formula	C23H24FN3O5S
Molecular weight	473.52 g/mol
Num. heavy atoms	33
Num. arom. heavy atoms	16
Fraction Csp3	0.30
Num. rotatable bonds	5
Num. H-bond acceptors	7
Num. H-bond donors	1
Molar Refractivity	130.23
TPSA	108.30 Å²

Lipophilicity	
Log $P_{o/w}$ (iLOGP)	2.84
Log $P_{o/w}$ (XLOGP3)	2.89
Log $P_{o/w}$ (WLOGP)	3.42
Log $P_{o/w}$ (MLOGP)	1.75
Log $P_{o/w}$ (SILICOS-IT)	2.18
Consensus Log $P_{o/w}$	2.61

Water Solubility	
Log *S* (ESOL)	-4.63
Solubility	1.12e-02 mg/ml ; 2.37e-05 mol/l
Class	Moderately soluble
Log *S* (Ali)	-4.82
Solubility	7.09e-03 mg/ml ; 1.50e-05 mol/l
Class	Moderately soluble
Log *S* (SILICOS-IT)	-5.69
Solubility	9.67e-04 mg/ml ; 2.04e-06 mol/l
Class	Moderately soluble

Pharmacokinetics	
GI absorption	High
BBB permeant	No
P-gp substrate	No
CYP1A2 inhibitor	No
CYP2C19 inhibitor	Yes
CYP2C9 inhibitor	Yes
CYP2D6 inhibitor	No
CYP3A4 inhibitor	Yes
Log K_p (skin permeation)	-7.14 cm/s

Druglikeness	
Lipinski	Yes; 0 violation
Ghose	No; 1 violation: MR>130
Veber	Yes
Egan	Yes
Muegge	Yes
Bioavailability Score	0.56

Medicinal Chemistry	
PAINS	0 alert
Brenk	0 alert
Leadlikeness	No; 1 violation: MW>350
Synthetic accessibility	3.35

Figure S2. Chemical and pharmacological properties of Compound **2** predicted by SwissADME.

Oral toxicity prediction results for input compound

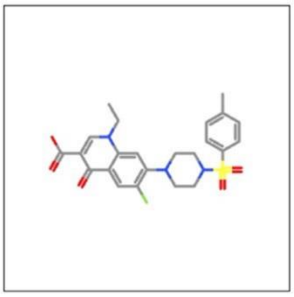

Predicted LD50: 2500mg/kg

Predicted Toxicity Class: 4

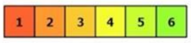

Average similarity: 76.87%

Prediction accuracy: 69.26%

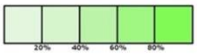

Name	C(C(O)=O)1=CN(CC)C2 (=O)C3=CC=C(C(=C3)C)
Molweight	473.52
Number of hydrogen bond acceptors	31
Number of hydrogen bond donors	1
Number of atoms	57
Number of bonds	60
Number of rotable bonds	5
Molecular refractivity	130.23
Topological Polar Surface Area	108.3
octanol/water partition coefficient(logP)	3.76

Toxicity Model Report

Classification	Target	Shorthand	Prediction	Probability
Organ toxicity	Hepatotoxicity	dili	Active	0.54
Toxicity end points	Carcinogenicity	carcino	Inactive	0.62
Toxicity end points	Immunotoxicity	immuno	Inactive	0.99
Toxicity end points	Mutagenicity	mutagen	Inactive	0.69
Toxicity end points	Cytotoxicity	cyto	Inactive	0.74
Tox21-Nuclear receptor signalling pathways	Aryl hydrocarbon Receptor (AhR)	nr_ahr	Inactive	0.94
Tox21-Nuclear receptor signalling pathways	Androgen Receptor (AR)	nr_ar	Inactive	0.88
Tox21-Nuclear receptor signalling pathways	Androgen Receptor Ligand Binding Domain (AR-LBD)	nr_ar_lbd	Inactive	0.85
Tox21-Nuclear receptor signalling pathways	Aromatase	nr_aromatase	Inactive	0.94
Tox21-Nuclear receptor signalling pathways	Estrogen Receptor Alpha (ER)	nr_er	Inactive	0.89
Tox21-Nuclear receptor signalling pathways	Estrogen Receptor Ligand Binding Domain (ER-LBD)	nr_er_lbd	Inactive	0.96
Tox21-Nuclear receptor signalling pathways	Peroxisome Proliferator Activated Receptor Gamma (PPAR-Gamma)	nr_ppar_gamma	Inactive	0.94
Tox21-Stress response pathways	Nuclear factor (erythroid-derived 2)-like 2/antioxidant responsive element (nrf2/ARE)	sr_are	Inactive	0.97
Tox21-Stress response pathways	Heat shock factor response element (HSE)	sr_hse	Inactive	0.97
Tox21-Stress response pathways	Mitochondrial Membrane Potential (MMP)	sr_mmp	Inactive	0.81
Tox21-Stress response pathways	Phosphoprotein (Tumor Supressor) p53	sr_p53	Inactive	0.96
Tox21-Stress response pathways	ATPase family AAA domain-containing protein 5 (ATAD5)	sr_atad5	Inactive	0.99

Figure S3. Toxicity prediction of Compound **2** by ProTox-II.

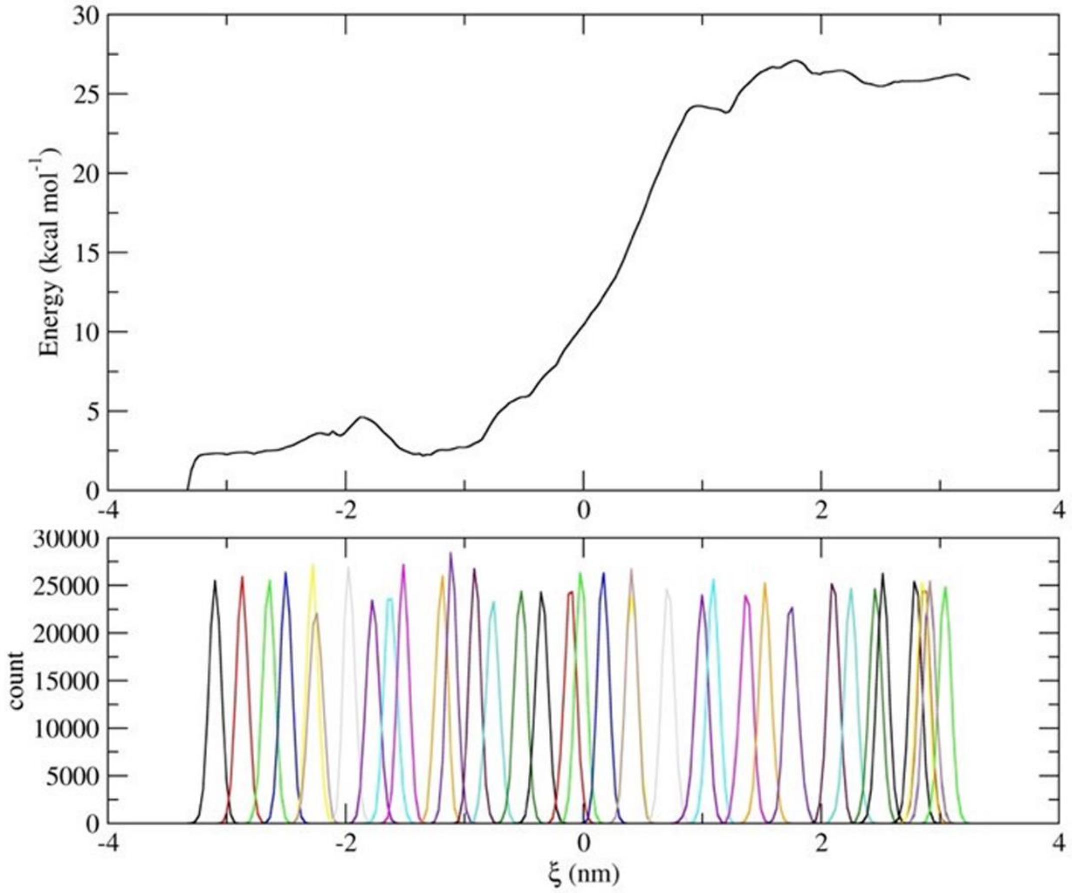

Figure S4. Free energy profile of Compound **2** for umbrella sampling
(Top) Z-axis energy gradient for the Gram-positive bacterial membrane model of Compound **2**. (Bottom) Sampling window at each reaction coordinate.

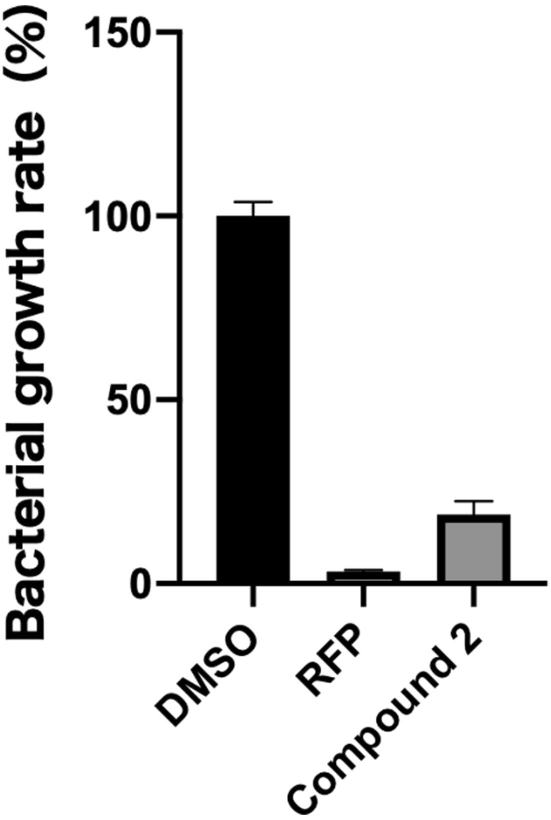

Figure S5. Growth inhibition rate against Mycobacterium
Negative control was 0.3% DMSO and positive control was 100 μM rifampicin (RFP). Concentration of Compound **2** is 100 μM.
